# Antiosteoporosis Effect of *Radix Scutellariae* Extract on Density and Microstructure of Long Bones in Tail-Suspended Sprague-Dawley Rats

**DOI:** 10.1155/2013/753703

**Published:** 2013-10-09

**Authors:** Chen-Rui Li, Guang-Wei Zhang, Yin-Bo Niu, Ya-Lei Pan, Yuan-Kun Zhai, Qi-Bing Mei

**Affiliations:** ^1^Key Laboratory for Space Biosciences & Biotechnology, School of Life Sciences, Northwestern Polytechnical University, Xi'an, Shaanxi, China; ^2^Xi'an Medical University, Xi'an, Shaanxi 710021, China; ^3^Department of Pharmacology, School of Pharmacy, The Fourth Military Medical University, Xi'an, Shaanxi, China

## Abstract

*Radix Scutellariae (RS)*, a medicinal herb, is extensively employed in traditional Chinese medicines and modern herbal prescriptions. Two major flavonoids in RS were known to induce osteoblastic differentiation and inhibit osteoclast differentiation, respectively. This study aimed to investigate the effect of *Radix Scutellariae* extract (RSE) against bone loss induced by mechanical inactivity or weightlessness. A hindlimb unloading tail-suspended rat model (TS) was established to determine the effect of RSE on bone mineral density and bone microarchitecture. Treatment of RSE at 50 mg/kg/day and alendronate (ALE) at 2 mg/kg/day as positive control for 42 days significantly increased the bone mineral density and mechanical strength compared with TS group. Enhanced bone turnover markers by TS treatment were attenuated by RSE and ALE administration. Deterioration of bone trabecula induced by TS was prevented. Moreover, both treatments counteracted the reduction of bone volume fraction, trabecular thickness and number, and connectivity density. In conclusion, RSE was demonstrated for the first time to prevent osteoporosis induced by TS treatment, which suggests the potential application of RSE in the treatment of disuse-induced osteoporosis.

## 1. Introduction

Mechanical load is critical to the maintenance of skeleton integrity. Unloading during space flight, immobilization, or prolonged bed rest would interrupt the bone metabolic balance between bone formation and resorption, resulting in loss of bone mineral, disruption of bone architecture, and impairment of bone mechanical properties [1–4]. Among 45 individual crew members after long-duration missions, the losses of bone mineral were up to 2%–9% [5]. After 17 weeks of horizontal bed rest, a 3.4% decrease of total hip bone mineral density (BMD) was observed in 18 control subjects [6]. Disuse-induced osteoporosis not only threatens the safety and health of the astronaut during space flight, but also increases the susceptibility to fractures in patients and elderly requiring bed rest. It is essential to find relevant countermeasures to reduce or prevent such bone loss.

A number of medicinal herbs or natural compounds show preventative effect on bone loss induced by physical inactivity or estrogen deficiency, such as Icaritin, Resveratrol, Astragaloside II, Epimedium, Radix Dipsaci, Eucommia ulmoides Oliv. cortex, Rhizoma Drynariae, herbal formulation of Epimedii Herba, Ligustri Lucidi Fructus and Psoraleae Fructus [7–15]. *Radix Scutellariae *(RS) is the dried root of *Scutellariae baicalensis* Georgi. It is officially listed in Chinese Pharmacopoeia with various pharmacological effects including anti-inflammation, antioxidation, neuro-protection, and immunomodulation [16–19]. Six flavones in RS have been reported to be the major bioactive components, namely, baicalein, wogonin, oroxylin A, baicalin, wogonoside, and oroxylin A-7-*O*-glucuronide [20, 21]. As the most abundant constituent in RS, baicalin was demonstrated to induce osteoblastic differentiation via Wnt/*β*-catenin signaling pathway [22]. Moreover, baicalein, the aglycone form of baicalin, inhibited osteoclast differentiation and induced mature osteoclast apoptosis [23].

Although *in vitro* evidence supported the osteoprotective effect of two individual flavones in RS, there is no direct evidence confirming their antiosteoporosis effect* in vivo*. Herbal extracts instead of the single component agent are frequently employed in proprietary traditional Chinese medicine products. Therefore, it is important to investigate the effect of *Radix Scutellariae* extract (RSE) on preventing bone loss* in vivo*. Hindlimb unloading tail-suspension (TS) model is extensively utilized as a simulated mechanical inactivity model under microgravity environment [24]. In rats, hindlimb unloading would result in a significant bone response including the loss of bone mineral and changes in calcium metabolism and biochemical markers of bone turnover, which are similar to the response in human [25]. Therefore, the current study aimed to determine the osteoprotective effect of RSE on the bone mineral density and characterize the microarchitecture of trabecular bone using TS rats.

## 2. Materials and Methods

### 2.1. Materials and Reagents

*Radix Scutellariae* extract (RSE) was purchased from Shanghai u-sea biotech co., Ltd. (Shanghai China). RSE was treated following our previous HPLC/UV method and quantified to contain 55.10 ± 1.76 of baicalin, 1.87 ± 0.01 of wogonoside, 4.04 ± 0.01 of oroxylin A-7-*O*-glucuronide, 1.43 ± 0.05 of baicalein, 0.38 ± 0.01 of wogonin, and 0.12 ± 0.00 of oroxylin A (mg/100 mg) [21]. Alendronate sodium (ALE) was supplied by Sigma-Aldrich Chem. Co. (Milwaukee, WI, USA). Distilled and deionized water was used throughout the experiment.

### 2.2. Animals and Treatments

Male Sprague-Dawley rats (SIPPR-BK Experimental Animal Ltd.) with the age of 8 weeks were supplied by the Animal Center of the Fourth Military Medical University (Xi'an, China). The rats were housed in an air-conditioned room (24°C) under a 12/12 h light/dark cycle. They were fed with standard rodent chow containing 0.9% calcium and 0.7% phosphate and had free access to water. The animals were treated under the approval by the Institutional Ethics Committee of the Fourth Military Medical University.

After one week of acclimatization to the keeping environment, rats were randomized into four groups: control group (CH, *n* = 6), tail-suspended group without treatment (TS, *n* = 6), and tail-suspended group treated with ALE and RSE (TS-ALE and TS-RSE, *n* = 6 each group), respectively. ALE and RSE were administrated to rats by intragastric gavage at a dose of 2 and 50 mg/kg body weights/day, respectively. Rats in CH and TS groups received equal volume of distilled water vehicle. Dosing lasted for 6 weeks and rats undertook tail-suspension from the third week. The procedures of tail-suspension followed the methods of Morey-Holton with modification [24].

One day before the sacrifice, rats were fasted for 12 h and the urine was collected for the other 12 h. At the end of tail-suspension, rats were sacrificed by exsanguination, and blood was collected by cardiac puncture. Serum was separated by centrifugation at 2,000 rpm for 20 min. Urine and serum samples were stored at −80°C for biochemistry evaluation. Femurs were dissected and stored in saline at −20°C for microCT assay and bone biomechanical tests.

### 2.3. Bone Histomorphometry Analysis

On day 29 and day 40, rats received calcein saline solution at 10 mg/kg BW by intramuscular injection to label bone formation surfaces. After sacrifice, right tibiae were removed and dehydrated in graded concentrations of ethanol (70%, 85%, 90%, and 100% graded ethanol). Embedded in methyl methacrylate without decalcification, frontal sections of proximal tibiae were sliced at 5 *μ*m. Bone formation was examined under fluorescence microscope (Leica image analysis system, Q500MC) with the excitation wavelength and emission wavelength at 490 and 515 nm, respectively.

### 2.4. Bone Mineral Density (BMD) Analysis

BMD at distal femoral metaphysic and proximal tibial metaphysic were measured by dual-energy X-ray absorptiometry (DXA) assay (Lunar Prodigy Advance DXA, GE healthcare, Madison, WI, USA). Data were calculated automatically by purpose-designed software (enCORE 2006, GE Healthcare, Madison, WI, USA). Before measurement, rats were anesthetized by and fixed at repeatable positioning. Total BMD was measured including the cross-sectional areas of both cortical and trabecular bones.

### 2.5. Microcomputed Tomography (MicroCT) Analysis

The microarchitecture of the left distal femur was analyzed by a desktop eXplore Locus SP Preclinical Specimen microCT (GE Healthcare, Madison, WI, USA). Femurs were aligned perpendicularly and scanned with a resolution of 12 *μ*m voxel. Three-dimensional images were acquired from microCT Evaluation Program (V5.0A). Trabecular bone was separated from the cortical bone by free drawing regions of interest with the MicroView program (GE healthcare, Madison, WI, USA) and a multiple Intel processor-based microCT workstation provided with the scanner. The volume of interest, 2 mm below the metaphyseal line, was chosen within 100 continuous slices. Morphologic measurements were performed and corresponding parameters were obtained including bone volume density (BV/TV), trabecular thickness (Tb. Th), trabecular number (Tb. N), trabecular separation (Tb. Sp), and connectivity density (Conn. D). Cortical thickness (Cr. Th) of femur was also analyzed and compared among various groups.

### 2.6. Biomechanical Test

Before measurement, rat left femurs were restored to room temperature. The bone strength was measured using a three-point bending test (MTS, Eden, Prairie, MN, USA). Bones were put on two supports spaced 20 mm apart, and load was applied to the middle of the bone at a deformation rate of 2 mm/min until fracture occurred. The central loading point was displaced and then the force and displacement data were recorded for further calculation. The inner and outer widths as well as the inner and outer height of the femur at the break point were also determined. A load-deformation curve could be plotted when a load is applied to the femoral midshaft. The femur displacement (*d*) increases linearly with applied load (*F*). The maximum slope of the curve (Δ*F*/Δ*d*) is titled stiffness which represents the structural rigidity of the bone. If the applied load is increased continuously, a yield point is reached and bone deforms nonelastically. When the maximum load is applied, bending further increases until a fracture occurs. Energy is the force that induces the fracture and is represented by the area under load-deformation curve [26]. Based on the initial linear part of the load deformation curve, Young's modulus (maximum slope of the stress-strain curve, *E*) and maximum stress (*F*_max⁡_/cross-sectional  area,  *σ*_max⁡_) were calculated as

(1) $$\begin{array}{c} E=\frac{\left({FL}^{3}\right)}{\left(d48I\right)},\\ \sigma_{\max}=\frac{\left(FLD\right)}{\left(8I\right)}, \end{array}$$

where *F* is the maximum load (ultimate strength,  *F*_max⁡_),  *L*  is the distance between supporting points, *d* is the displacement, *I* is the moment of inertia of the cross-section in relation to the horizontal axis (electronic supplementary material, ESM), and *D* is the outer height of femur.

### 2.7. Urine and Serum Biochemistry Measurements

Calcium in serum and urine (S-Ca and U-Ca) and phosphorus in serum and urine (S-P and U-P) as well as urinary creatinine (Cr) were quantified by an automatic biochemical analyzer (Cobas Integra 400 plus, Roche diagnostics, Basel, Switzerland) using the original kits from Roche Diagnostics (IN, USA). Serum osteocalcin (OC) was measured by a rat OC ELISA kit (Biomedical Technologies, Stoughton, MA, USA). Urinary deoxypyridinoline (DPD) was assayed using a rat DPD ELISA kit (Quidel Corp., San Diego, CA, USA). Serum alkaline phosphatase (APL), titrate resistant acid phosphatase (TRACP), urine type I collagen carboxy-terminal peptide (CTx), and type I collagen amino-terminal peptide (NTx) were quantified by ELISA kit (Beijing Sino-Uk Institute of Biological Technology, China).

### 2.8. Statistical Analysis

Data are presented as mean ± SD. All the statistical analyses were performed with the Statistical Package of Social Science (SPSS, Version 16.0, USA). Comparisons of group variance were performed by one-way ANOVA followed by Tukey's multiple comparison tests. Statistical difference was considered significant at  *P* < 0.05.

## 3. Results

### 3.1. Body Weights

**Figure 1:**
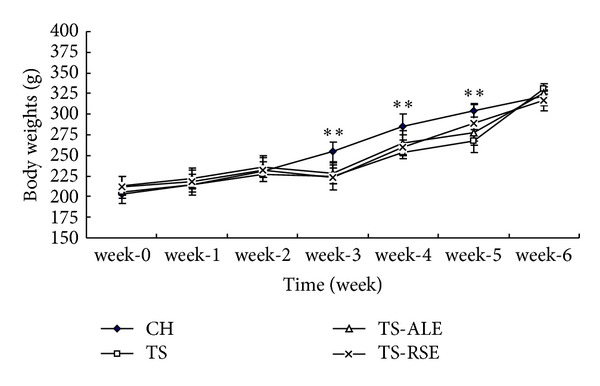
Measurements of rat body weights within various treatment groups. Values are presented by means ± SD, *n* = 6. *versus CH group: *P < 0.05, **P < 0.01.

After a 6-week administration, there was no significant difference in the initial and final body weights among all treatment groups. The trends of body weights changes in all groups were plotted versus time (Figure 1). It was found that the body weights of rats in all groups showed similar trends of increase during the first three weeks. Once the tail-suspension started (the third week), the body weights in TS, TS-ALE, and TS-RSE were significantly decreased compared with CH group in the fourth week, while they recovered with time. In the sixth week, the body weights in all groups were similar without significant difference.

### 3.2. Bone Histomorphometry Analysis

**Figure 2:**
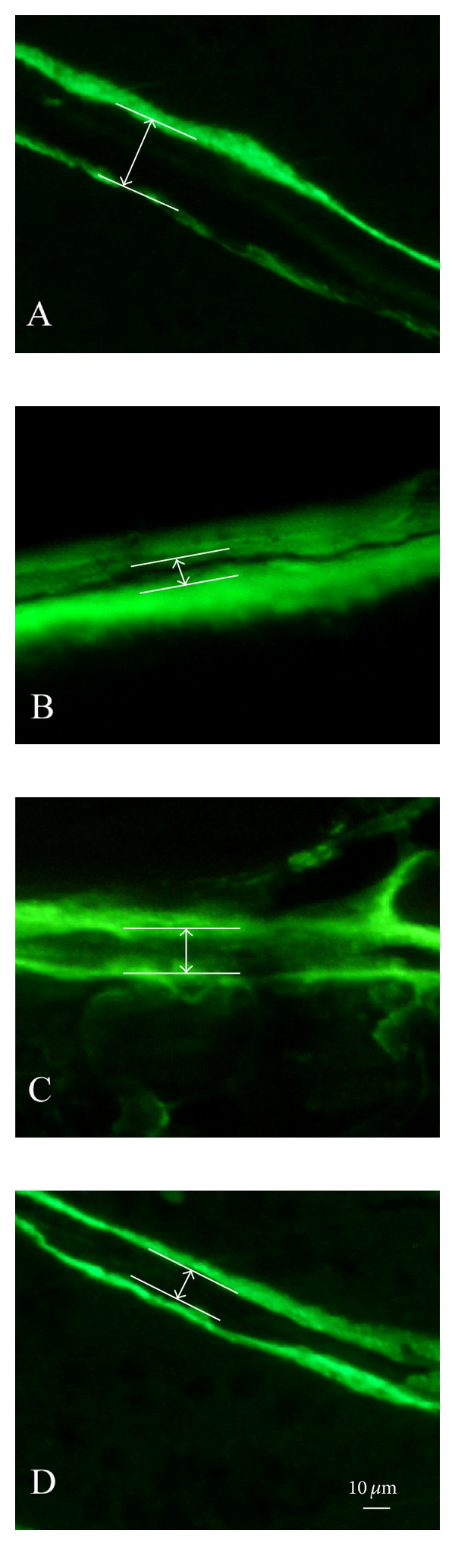
Representative fluorescence micrographs of trabecular bone sections showing green calcein labels within various treatment groups: (A) CH, (B) TS, (C) TS-ALE, and (D) TS-RSE.

Representative fluorescent micrographs of the nondecalcified trabecular bone sections within various treatment groups are shown in Figure 2. Bone formation was examined by sequential labels with fluorescent dye in nondecalcified bone trabecular sections. Compared with CH rats, the growth of new bone has been significantly suppressed in TS rats with a narrow width between the two labeling bands in the rats treated by TS. The width between two green sequential labels was much broader in ALE and RSE treated rats than in TS rats.

### 3.3. BMD Evaluation

Results of BMD changes after various treatments were shown in Table 1. When compared with CH rats, tail-suspension induced significant loss of BMD in both distal femur (*P* < 0.001) and proximal tibia (*P* < 0.01). Rats with the treatment of RSE (*P* < 0.05) had increased BMD values compared with TS rats. ALE exhibited stronger effect on the prevention of BMD loss than that exhibited by RSE.

### 3.4. Bone Microarchitecture

**Figure 3:**
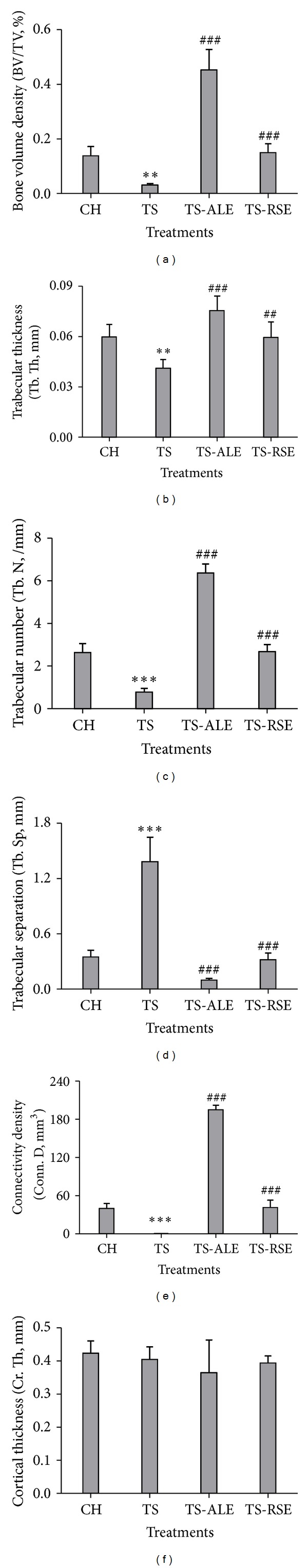
Measurements of rat femur within various treatment groups: (a) bone volume density (BV/TV), (b) trabecular thickness (Tb. Th), (c) trabecular number (Tb. N), (d) trabecular separation (Tb. Sp), (e) connectivity density (Conn. D), and (f) cortical thickness (Cr. Th). *versus CH group: *P < 0.05, **P < 0.01, and ***P < 0.001; ^#^versus TS group: ^#^ *P* < 0.05, ^##^ *P* < 0.01, and ^###^ *P* < 0.001.

**Figure 4:**
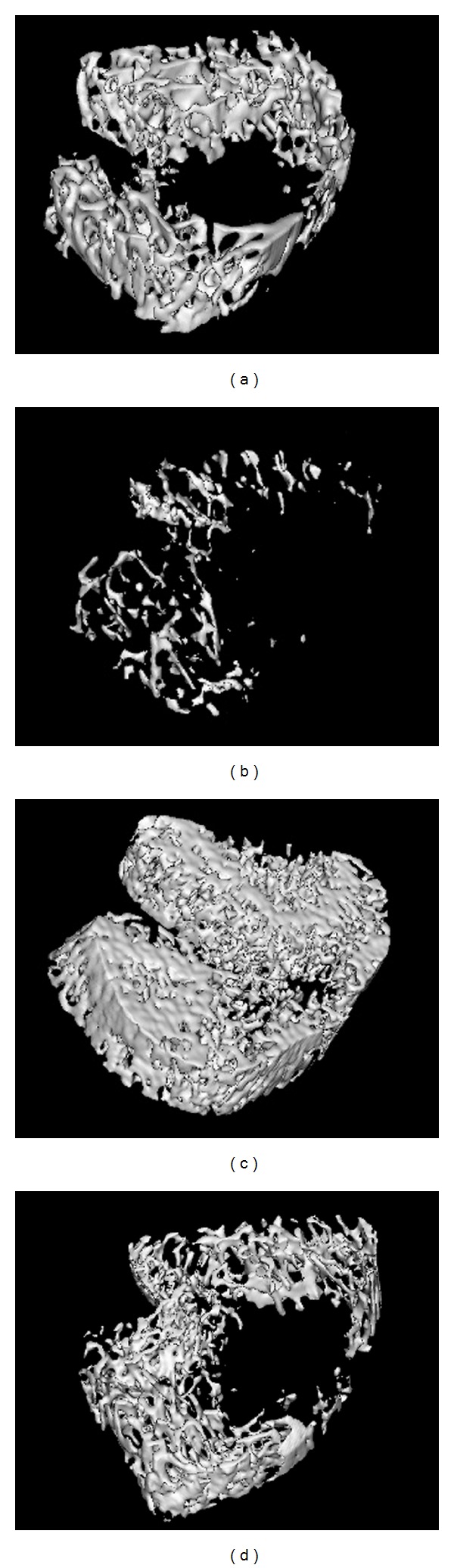
Representative 3D images of trabecular bone within the distal femoral metaphysic region in various treatment groups: (a) CH, (b) TS, (c) TS-ALE, and (d) TS-RSE.

Trabecular microarchitecture of rat femur was analyzed by microCT and the results were shown in Figure 3. The representative 3D images of trabecular bone are shown in Figure 4. Compared with CH rats, tail-suspension could significantly decrease the bone volume fraction (BV/TV) (−77.6%, *P* < 0.01), trabecular thickness (Tb. Th) (−31.2%, *P* < 0.01), trabecular number (Tb. N) (−70.4%, *P* < 0.01), and connectivity density (Conn. D) (−99.1%, *P* < 0.001), whereas a significant higher trabecular separation (Tb. Sp) (295.0%, *P* < 0.001) was observed in TS rats. Compared with TS rats, all the microarchitecture parameters were significantly improved by ALE and RSE treatment (*P* < 0.01) and TS-RSE rats had comparable parameters with CH rats. Moreover, TS or RSE and ALE treatment showed no significant effect on the cortical thickness.

### 3.5. Biochemistry Assay

Results of serum and urinary biochemistry measurements are shown in Table 2. After TS treatment, serum levels of Ca and P have not been altered, whereas their urinary levels have been significantly enhanced (*P* < 0.001 or *P* < 0.05). Compared with TS rats, the treatment of ALE and RSE could significantly suppress the TS-induced elevation in the serum concentration of TRACP (*P* < 0.001) and the urinary levels of Ca/Cr, P/Cr, DPD/Cr (*P* < 0.001), CTx (*P* < 0.01), and NTx (*P* < 0.001). The TS-induced rise in serum levels of ALP (*P* < 0.001) was prevented by the ALE and RSE administration.

### 3.6. Biomechanical Test

The biomechanical parameters of three-point bending test on femur were summarized in Table 3. TS treatment significantly decreased the maximum load and stiffness (*P* < 0.001) as well as the maximum stress (*P* < 0.05) and Young's modulus (*P* < 0.01) with CH rats, whereas the energy was not influenced by TS treatment. The administration of ALE and RSE could significantly prevent the TS-induced decrease in maximum load and maximum stress (*P* < 0.05). Young's modulus (*P* < 0.001 or *P* < 0.01) and stiffness (*P* < 0.001 or *P* < 0.05) were also enhanced by ALE and RSE treatments.

## 4. Discussion

Due to the difficulty and high cost of conducting experiments during a space flight, a number of *in vitro* and *in vivo* ground-based models have been established to simulate the aerospace environment and conditions during a space mission [25]. TS rat/mice model is one of the frequently employed animal models [27]. In this study, TS treatment generated a significant decrease of BMD in femur and tibiae (−28.6% and −24.3%, resp.). MicroCT assay also demonstrated a deteriorated condition of trabecular femur after TS treatment, which could be reflected directly from the 3-D images or the corresponding architectural parameters. The trend of changes in BMD and femur trabecular properties was comparable with previous studies, which demonstrated that the established TS rat model could be used for further study [9, 12, 28]. Decreased values of biomechanical parameters also indicated the deduced strength and stiffness of femur by TS treatment. The TS-induced bone loss was coupled with accelerated bone remodeling which was evidenced by increased bone turnover markers in serum and urine such as ALP, DPD, TRACP, CTx, and NTx. The serum level of OC also showed tendency of increase, although the difference was not significant enough.

Mechanical load is crucial for the maintenance of bone mass and strength, and physical inactivity would accelerate the bone microarchitecture deterioration and demineralization [3, 29]. The potential mechanisms behind the weightlessness-induced bone loss have not been fully understood, but it is suggested that the increased inflammation and production of oxygen-derived free radicals might be contributory in part [30–32]. Celecoxib, a selective cyclooxygenase-2 (COX-2) inhibitor, could suppress the restoration of tibial trabecular bone formation and the acute recovery of trabecular bone in a TS C57BL/6j mice model, which implied the important role of COX-2 in acute bone adaptation during reloading after unloading [33]. *Radix Scutellariae *(RS) has a long history of application in traditional Chinese medicine to clear away heat and purge fire [34]. Both RSE and baicalin, which are the most abundant flavone in RS, show strong pharmacological effect of anti-inflammation and antioxidation [16, 17, 35–37]. Therefore, it is of high possibility that RSE is capable of preventing weightlessness-induced bone loss.

In the present study, the treatment of RS for 42 continuous days (50 mg/body weight/day, b.i.d.) before and during tail-suspension could effectively attenuate the deterioration in femoral and tibiae trabecular bones. RSE treatment could restore the femur and tibia BMD compared with TS rats. The microarchitectural properties were also improved after the RSE administration including the increased BV/TV, Tb. Th, Tb.N, and Conn. D, whereas the trabecular separation decreased. As reported, neither TS nor drug treatment influenced the cortical thickness [8]. Similar results were generated according to the biomechanical assay via a three-point bending test. TS treatment significantly decreased bone stiffness and made the femurs more fragile. ALE and RSE treatment showed a comparable effect on bone strength. Maximum load, stiffness, and energy reflect the structural biomechanical parameters which would be influenced by the shape and size of the bone. The actual effect of the treatment on biomechanical competence could be fully evaluated with resort to the geometric properties reflecting the material biomechanical parameters. Therefore, maximum stress and Young's modulus are corrected and generated [26].

With regard to bone turnover markers, RSE could decrease urinary Ca and P. The enhanced levels of bone resorption markers induced by TS were counteracted by RSE treatment. In particular, the increased TRACP and CTx were reversed to the level even comparable with CH group. Based on the results, RSE could reduce TS-induced bone remodeling, but so far it is hard to determine which dominated between the effect of RSE treatment on bone resorption and bone formation. Notably, *in vitro* study suggested that baicalin could promote osteoblast differentiation via Wnt/*β*-catenin signaling pathway, whereas its aglycone form baicalein inhibited osteoclast differentiation in another study [22, 23]. Although baicalin is the most abundant component, other constituents in RSE might also contribute via different mechanisms. Further experiments are warranted to reveal the potential mechanisms behind the osteogenetic effect of RSE as a flavonoid mixture.

As a classic antiresorptive biphosphonate, alendronate sodium (ALE) has been frequently prescribed in clinics for the prevention and treatment of postmenopausal osteoporosis [38]. Its role to inhibit normal bone resorption was also confirmed in tail-suspended rats, which resulted in the net bone mass increase [39]. Therefore, ALE was chosen as the positive control in the present study, and the dose was established based on a previous study by Qi et al. [40]. As expected, the TS-induced loss of bone mineral density and decrease of bone strength and stiffness were prevented by ALE treatment, which was similar to the literature report [41]. Based on the microCT result, massive bone trabeculae were observed in ALE treatment group which were even denser than those of the CH group. Based on further ANOVA analysis, ALE treatment increased bone volume density and trabecular bone number compared with CH rats. The effect could be reflected based on the 3-D images. In the study by Qi et al., ALE at 2.0 mg/kg/day could also significantly reverse the decreased trabecular parameters induced by tail-suspension, such as BV/TV, Tb. N, Tb.Th, and Conn.D. ALE treatment showed a stronger effect on the increase of trabecular parameters in our study than that in Qi's study. Six-month-old rats were employed in Qi's work, whereas we used younger rats at 2 months old. Rat age might be a factor that induces the different results, and rats at different ages might respond differently to the ALE treatment.

## 5. Conclusion

The current study firstly demonstrated the *in vivo* osteoprotective effect of RSE. It could effectively reverse the deteriorated condition of bone trabecular in femur and tibiae induced by TS treatment. The results suggested the therapeutic effect of RSE as an alternative supplement to be applied in the prevention and treatment of disuse osteoporosis induced by mechanical inactivity.

## Figures and Tables

**Table 1:** Bone mineral density of distal femur and proximal tibia within various treatment groups (g/cm^2^, *n* = 6).

Item	Treatments			
	CH	TS	TS-ALE	TS-RSE
fBMD	0.21 ± 0.01	0.15 ± 0.01***	0.18 ± 0.01^##^	0.17 ± 0.01^#^
tBMD	0.15 ± 0.02	0.12 ± 0.00**	0.15 ± 0.01^##^	0.14 ± 0.02^#^

fBMD: femur bone mineral density; tBMD: tibia bone mineral density.

*versus CH group: *P < 0.05, **P < 0.01, and ***P < 0.001.

^#^versus TS group: ^#^ *P* < 0.05, ^##^ *P* < 0.01, and ^###^ *P* < 0.001.

**Table 2:** Biochemical parameters in rat urine and serum within various treatment groups.

Parameters	Treatments			
	CH	TS	TS-ALE	TS-RSE
S-Ca (mM)	2.43 ± 0.18	2.34 ± 0.08	2.42 ± 0.22	2.35 ± 0.19
S-P (mM)	1.68 ± 0.05	1.71 ± 0.08	1.64 ± 0.16	1.66 ± 0.14
OC (pg/mL)	0.21 ± 0.02	0.23 ± 0.03	0.20 ± 0.01	0.20 ± 0.03
ALP (*μ*g/L)	191.89 ± 13.76	275.98 ± 20.11***	196.66 ± 3.50^###^	197.80 ± 15.96^###^
TRACP (pg/L)	2351.54 ± 257.77	3178.12 ± 196.41***	2368.72 ± 461.65^##^	2390.20 ± 456.81^##^

U-Ca/Cr (mM/mM)	0.57 ± 0.06	1.58 ± 0.19***	0.70 ± 0.15^###^	0.95 ± 0.05^###^
U-P/Cr (mM/mM)	5.54 ± 0.48	8.11 ± 1.45*	6.22 ± 0.29^#^	5.90 ± 0.68^#^
DPD/Cr (mM/mM)	0.15 ± 0.05	0.43 ± 0.05***	0.26 ± 0.03^###^	0.25 ± 0.04^###^
CTx (*μ*g/L)	4.72 ± 0.33	5.60 ± 0.19**	4.63 ± 0.22^###^	4.68 ± 0.36^##^
NTx (nM)	14.96 ± 0.86	19.29 ± 0.36***	15.39 ± 2.45^##^	15.95 ± 2.35^#^

S-Ca: serum Ca; S-P: serum phosphorus; OC: osteocalcin; ALP: alkaline phosphatase; TRACP: tartrate resistant acid phosphatase; U-Ca: urinary Ca; U-P: urinary phosphorus; CTx: type I collagen carboxy-terminal peptide; NTx: type I collagen aminoterminal peptide; DPD: deoxypyridinoline; Cr: creatinine.

*versus CH group: *P < 0.05, **P < 0.01, and ***P < 0.001.

^#^versus TS group: ^#^ *P *<0.05, ^##^ *P* < 0.01, and ^###^ *P* < 0.001.

**Table 3:** Effect of RSE treatment on bone biomechanical parameters in rat femoral diaphysis.

Parameters	Treatments			
	CH	TS	TS-ALE	TS-RSE
Maximum stress (MPa)	165.27 ± 16.46	137.08 ± 9.93*	156.29 ± 14.95^#^	159.29 ± 14.46^#^
Young's modulus (MPa)	3478.33 ± 723.17	2130.25 ± 375.55**	3610.74 ± 269.63^###^	2955.78 ± 401.10^##^
Maximum load (N)	110.82 ± 9.22	75.99 ± 6.60***	86.73 ± 6.44^#^	85.70 ± 6.02^#^
Stiffness (N/mm)	155.95 ± 29.28	77.75 ± 13.82***	132.49 ± 15.31^###^	101.12 ± 18.05^#^
Energy (N × m)	0.07 ± 0.01	0.08 ± 0.03	0.07 ± 0.03	0.07 ± 0.03

*versus CH group: *P < 0.05, **P < 0.01, and ***P < 0.001.

^#^versus TS group: ^#^ *P* < 0.05, ^##^ *P* < 0.01, and ^###^ *P* < 0.001.
